# Comparison Study between RMS and Edge Detection Image Processing Algorithms for a Pulsed Laser UWPI (Ultrasonic Wave Propagation Imaging)-Based NDT Technique

**DOI:** 10.3390/s17061224

**Published:** 2017-05-26

**Authors:** Changgil Lee, Aoqi Zhang, Byoungjoon Yu, Seunghee Park

**Affiliations:** 1 School of Civil, Architectural Engineering and Landscape Architecture, Sungkyunkwan University, Gyeonggi-do, Suwon-si 16419, Korea; tolck81@gmail.com (C.L.); zhangaoqi623@hotmail.com (A.Z.); 2 Department of Convergence Engineering for Future City, Sungkyunkwan University, Gyeonggi-do, Suwon-si 16419, Korea; mysinmu123@naver.com

**Keywords:** pulsed laser scanning, ultrasonic waves, plate-like structures, crack, corrosion, edge detection

## Abstract

In this study, a non-contact laser ultrasonic propagation imaging technique was applied to detect the damage of plate-like structures. Lamb waves were generated by an Nd:YAG pulse laser system, while a galvanometer-based laser scanner was used to scan the preliminarily designated area. The signals of the structural responses were measured using a piezoelectric sensor attached on the front or back side of the plates. The obtained responses were analyzed by calculating the root mean square (RMS) values to achieve the visualization of structural defects such as crack, corrosion, and so on. If the propagating waves encounter the damage, the waves are scattered at the damage and the energy of the scattered waves can be expressed by the RMS values. In this study, notch and corrosion were artificially formed on aluminum plates and were considered as structural defects. The notches were created with different depths and angles on the aluminum plates, and the corrosion damage was formed with different depths and areas. To visualize the damage more clearly, edge detection methodologies were applied to the RMS images and the feasibility of the methods was investigated. The results showed that most of the edge detection methods were good at detecting the shape and/or the size of the damage while they had poor performance of detecting the depth of the damage.

## 1. Introduction

Non-destructive testing (NDT) technology has been used for a number of decades, and NDT techniques have been successfully applied in many practical applications in various fields such as civil, mechanical, and aerospace engineering, etc. [1]. These methods can prolong the lifetimes of structures, and facilitate maintenance of structural health to minimize premature part changes. The method that can easily generate understandable detection results is preferred. With these methods, the cost of training personnel can be reduced, and the risk of human error can be decreased. In this case, the methods for damage detection with imaging capabilities have great potential to fulfill these requirements. Multiple locations on a target structure, which potentially contain various types of damage, can be monitored at the same time by using these methods. The location and degree of damage is very important for making a decision about a maintenance plan [2]. For example, due to the restricted accessibility of some structures such as nuclear power plants and due to the high-precision geometries or other inaccessible parts of a structure, the detection of structural safety is particularly difficult. Therefore, when damage detection is needed in a large-scale structure, it is necessary to develop a non-contact NDT method for damage detection [3].

In NDT, the methods for damage detection should be effective and also have high throughput, because of the increasing size of structures which need to be inspected. Ultrasonic waves are sensitive to most material damage and are not radiation hazards. In addition, they also can provide many features for damage characterization. Therefore, a wide range of inspection methods based on acoustic-ultrasonic waves have imaging capabilities. Not only that, most laser-ultrasonic systems can be integrated into mobile systems, and the laser also provides non-contact remote characteristics. To achieve that goal, some acoustic and ultrasonic wave technologies have been developed, such as full-field laser wave field imaging, laser vibrometry, laser interferometry, and pulsed lasers. As one of the full-field laser wave field imaging techniques, the holography based imaging technique requires a highly diffusive surface of the target structure. However, holography is always regarded as a technique which requires dark rooms. Therefore this method is inappropriate for remote automatic detection, even though it has the capability of noncontact detection [4]. A previous study [5] showed that Lamb waves were generated in an aluminum plate immersed in water. The laser vibrometer was used to scan the target surface, in which the laser beam was perpendicular to the surface. The scan locations can be changed by moving the laser head. It was verified that Lamb waves can be propagated in an aluminum plate, in the previous study [6]. The result was confirmed by using a laser scanning vibrometer, and the propagating waves were visualized in the vicinity of flaw area. Their results showed that the effectiveness of flaw detection depends on the flaw size. Other studies [7,8,9] have used Lamb waves to detect the damage of aluminum plates by using a laser scanning vibrometer to scan the surface of the target side. Due to their low noise and narrow line widths (on the order of a few millihertz), a single-mode HeNe laser is the preferred light source for the laser Doppler vibrometers (LDVs). The flaws were measured by finding the areas with bigger signal values. However, laser scanning vibrometers still have some disadvantages, such as the limiting factor about capturing a full field, and the signal-to-noise ratio of the photodetector output [10,11]. To overcome these drawbacks, an Nd:YAG pulse laser system has been developed to generate the ultrasonic waves. This pulsed laser could provide many advantages such as fast wave generation with low pulse energy, good detection capability in complex structures, and a high spatial resolution [12,13].

In this study, a non-contact laser ultrasonic wave propagation imaging (UWPI) method using a Nd:YAG pulsed laser system was used to detect the damage on aluminum plates. An Nd:YAG pulse laser was used to generate the ultrasonic waves, and the laser scanner based on a galvanometer was used to scan the target structure. In order to measure the wave responses at this stage, a piezoelectric sensor was installed to the central position on the front or the back side of the scanned surface. The damage can be visualized by obtaining root mean square (RMS) images [14]. Additionally, a series of edge detection methodologies were applied to the RMS images and compared to improve the performance of the damage visualization. To verify the feasibility of the approach, aluminum plates with notch and corrosion damages were tested. In the case of notch damage, different depths and angles were considered while different area and depths were investigated for the corrosion damage.

## 2. Ultrasonic Wave Propagation Imaging (UWPI) System

**Figure 1:**
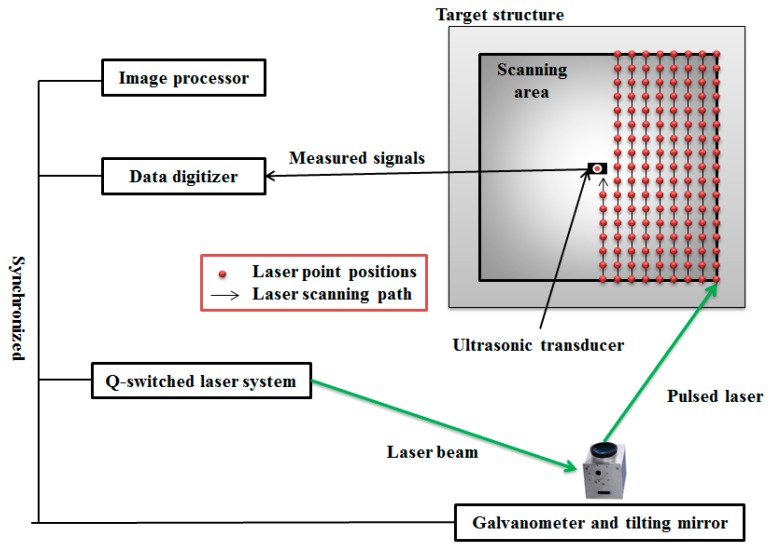
A schematic diagram of the laser-induced ultrasonic wave propagation imaging (UWPI) system.

As shown in Figure 1, the UWPI system includes an image processor, a high-speed data digitizer, a Q-switch pulsed laser system, a laser mirror scanner based on a galvanometer, and an ultrasound transducer. In this study, a Q-switch diode-pumped high-power solid-state Nd:YAG pulse laser [15] is used, with the wavelength of 532 nm and the maximum pulse repetition rate of 20 Hz. The laser mirror scanner can adjust the scanning location of the target structure, which is designed so that the laser beam can be reflected at the tilting mirrors in the scanner with the wavelength of 532 nm. For ensuring that the laser beam can efficiently scan the two dimensional area of the target structure, the tilting mirrors are designed so that the operating angles are orthogonal to each other. An f-theta lens is installed at the end of the laser scanner system so that the laser beam can be focused on the target area. In this study, the laser beam vertically scans the target structure in the horizontal direction along the scanning coordinate as shown in Figure 1, which is preliminarily designed at the image processor. Also, the measured wave signals from the ultrasonic sensor were saved and treated to obtain ultrasonic wave propagation images (UWPI). The details of the process for the UWPI are explained in the following section.

**Figure 2:**
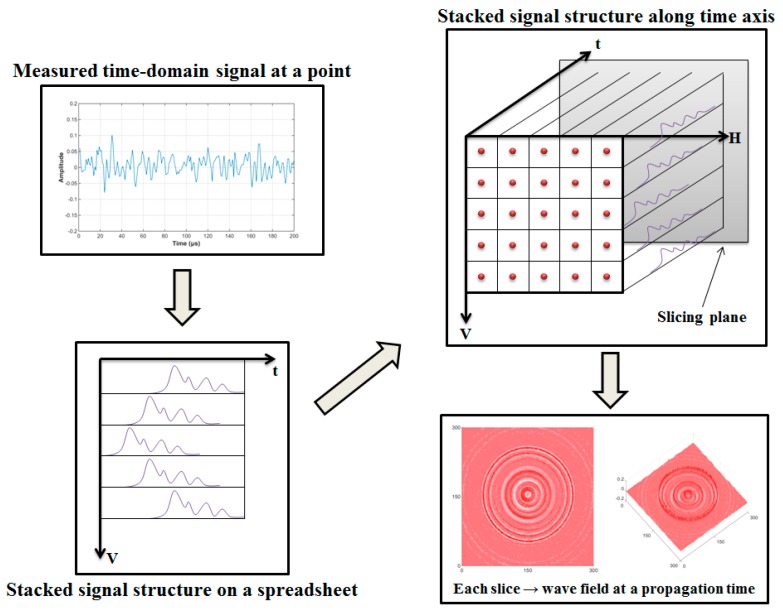
Ultrasonic wave propagation imaging process.

During the scanning process, due to the thermoelastic mechanism, the ultrasonic waves are generated at the point where the laser beam is impinged on the surface of the target structure and propagated to the ultrasonic sensor. An ultrasonic transducer which is installed at the front or the back side of the scanned surface measures the wave responses. In this study, an acoustic emission sensor (AE sensor) which is made of lead zirconate titanate piezoelectric ceramics is selected as an ultrasonic transducer. The imaging process of ultrasonic wave propagation is shown in Figure 2. Firstly, the time-domain signal is also obtained at each laser scanning point. In addition, a band-pass digital filter is used to filter the noise signals and improve the signal-to-noise ratio. After that, the filtered signal groups in a vertical structure on a spreadsheet can be stacked, for each laser scanning point on the vertical axis. Then, the stacked vertical data need to be stacked repeatedly along the horizontal axis. Finally, 3-D UWPI data can be obtained with the three axes of the vertical scan, horizontal scan, and time frame [16]. A snapshot of the ultrasonic wave propagation image can be captured by slicing the 3-D data at a certain time point. Using these snapshots, post image processing for damage detection is performed.

## 3. Imaging Process Algorithm for Damage Detection

### 3.1. Visualization Method Using Root Mean Square (RMS)

The propagating characteristics of ultrasonic waves can be more clearly expressed using the RMS images of the wave signals, because the RMS images can describe the energy distribution of the signals. The equation is shown in [17]:

(1) $$w_{RMS}(x,y)={\left[\frac{1}{N}\sum_{i=1}^{N}{\left(w_{i}(x,y,t)\right)}^{2}\right]}^{\frac{1}{2}}$$

where *N* is the number of signal samples, and *w*(*x*, *y*, *t*) are the reflected signals.

Because of more frequent accumulations of the standing wave energy at the location of the sensor, the bigger RMS values will be produced in this area. This makes it hard to detect damage far from the sensor. In this case, multiplying a weighting parameter can equalize the RMS value of entire area, as follows:

(2) $$w_{RMS}(x,y)\_W^{p}={\left[\frac{1}{N}\sum_{i=1}^{N}{\left(w_{i}(x,y,t)\right)}^{2}\cdot t^{p}\right]}^{\frac{1}{2}}$$

where *p* is the weighting parameter and *w_RMS_*(*x*, *y*)_*W^p^* is the weighted RMS function.

In this study, the weighting parameter was 2.

### 3.2. Edge Detection Method

Edge detection techniques are very popular and essential image preprocessing steps, especially in the areas of feature detection and feature extraction. In an image, the quantity of data significantly reduces at an edge area, but these data still retain basic information of the objects in the area. If the value of a pixel point exceeds a designated threshold, that point is declared as an edge location. Therefore, the edges have the higher pixel intensity values than the surrounding points. In this way, the edges can be detected by comparing the gradient value to the threshold value, and when the 1st derivative is the maximum, the 2nd derivative will be 0. This characteristic can be used for computer vision and image processing. The method has major features for a good ability to create the exact edge line. Therefore, edge detection is an active research area for better facilitating the image analysis. Nowadays, edge detection is usually used for object detection such as medical image processing, biometrics, and advanced computer imaging techniques [18]. Not only that, this method can also be used in SHM (Strutural Health Monitoring). In this study, the Sobel, Prewitt, Roberts, and Laplacian of Gaussian (LoG) operators are used to filter the images of the testing results.

#### 3.2.1. Sobel Operator

The Sobel edge detection technique was proposed by Sobel in 1970 [19]. The method is a spatial domain gradient-based edge detector. The Sobel operator performs a 2-D spatial gradient measurement on an image which consists of two gradient masks of size 3 × 3, one for horizontal changes, and another for vertical changes. In general, it is used to calculate the approximate absolute gradient magnitude (edge strength) at each single pixel point. The actual Sobel masks are as follows:

(3) $$Gx_{\left(sobel\right)}=\left[\begin{array}{ccc} -1 & 0 & +1\\ -2 & 0 & +2\\ -1 & 0 & +1 \end{array}\right]\;\mathrm{and}\;Gy_{\left(sobel\right)}=\left[\begin{array}{ccc} +1 & +2 & +1\\ 0 & 0 & 0\\ -1 & -2 & -1 \end{array}\right]$$

where *Gx* and *Gy* are the gradient component at each point that contain the horizontal and vertical direction. The gradient magnitude can be calculated using the formula:

(4) $$\left|G\right|=\sqrt{Gx^{2}+Gy^{2}}$$

Then, the approximate absolute gradient magnitude can be calculated using:

(5) |G|=|Gx|+|Gy|

Finally, using this information, the gradient direction *θ* is given by:

(6) θ=arctan(GyGx)

where in this case, *θ* = 0 means the direction of maximum contrast from the color of black to white runs from left to right on the image, and other angles can be measured anti-clockwise from it.

**Figure 3:**
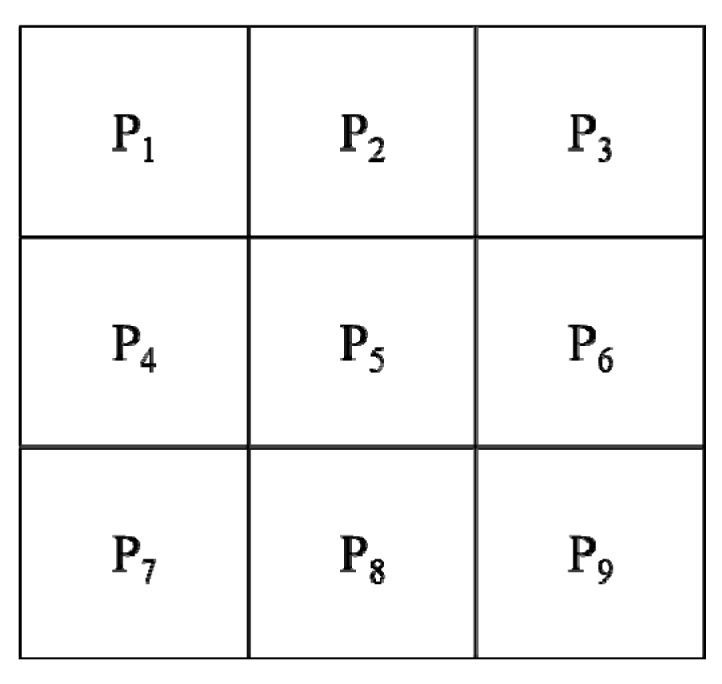
Pseudo-convolution masks for the Sobel operator used to quickly compute the approximate gradient magnitude.

In general, the absolute magnitude is the output that only the researchers can observe. Figure 3 shows that by using the pseudo-convolution operator, the two components of the gradient could be conveniently computed and added in a single pass over the input image.

Using this mask, the equation of approximate magnitude is given by:

(7) $$\left|G\right|=\left|\left(P_{1}+2\times P_{2}+P_{3})-(P_{7}+2\times P_{8}+P_{2}\right)\right|+\left|\left(P_{3}+2\times P_{6}+P_{2})-(P_{1}+2\times P_{4}+P_{7}\right)\right|$$

#### 3.2.2. Roberts Cross Operator

The Roberts cross operator has a good ability to perform a simple, quick calculation and 2-D spatial gradient measurement on an image [20]. The Roberts cross operator for the input is a grayscale image, as is the output. Pixel values of each point in the output data are the estimated absolute magnitude of the spatial gradient at that point. This operator consists of a pair of 2 × 2 convolution kernels as follows:

(8) $$Gx_{\left(Robert\right)}=\left[\begin{array}{cc} +1 & 0\\ 0 & -1 \end{array}\right]\;\mathrm{and}\;Gy_{\left(Robert\right)}=\left[\begin{array}{cc} 0 & +1\\ -1 & 0 \end{array}\right]$$

where one kernel is simply rotated by 90° to the other, and this mask is very similar to the Sobel operator.

The kernels are designed to maximize the response to the edges running at 45° to the pixel grid, one kernel will correspond to each of the two perpendicular orientations. These kernels are applied separately to form gradient components in each orientation (*Gx* and *Gy*). Therefore, the gradient magnitude can be defined as:

(9) $$\left|G_{\left(Robert\right)}\right|=\sqrt{Gx_{\left(Robert\right)}^{2}+Gy_{\left(Robert\right)}^{2}}$$

The approximate magnitude can be calculated by:

(10) $$\left|G_{\left(Robert\right)}\right|=\left|Gx_{\left(Robert\right)}\right|+\left|Gy_{\left(Robert\right)}\right|$$

The direction of the gradient (relative to the pixel grid orientation) is given by:

(11) θ=arctan(Gy(Robert)Gx(Robert))−3π4

When *θ* = 0, it has same characteristics as the Sobel operator.

**Figure 4:**
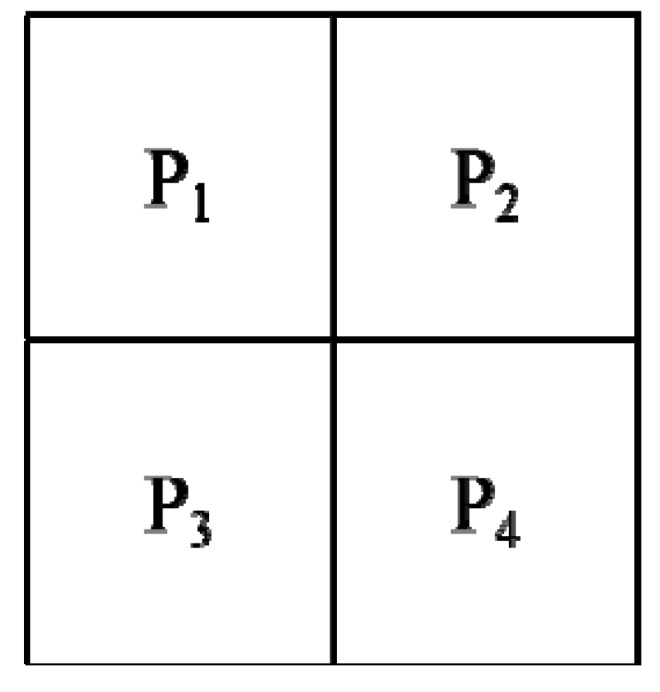
Pseudo-convolution masks for the Robert operator.

Not only that, the absolute magnitude is the output that only the researchers can observe. A pseudo-convolution operator is used to computed the gradient components and add in a single pass over the input image, as shown in Figure 4.

The approximate magnitude can be given by:

(12) $$\left|G_{\left(Robert\right)}\right|=\left|\left(P_{1}-P_{4}\right)\right|+\left|P_{2}-P_{3}\right|$$

#### 3.2.3. Prewitt Operator

The Prewitt operator [21] is similar to the Sobel operator. This operator can be used for detecting vertical and horizontal edges of the images. The Prewitt operator kernel is given by:

(13) $$Gx_{\left(Prewitt\right)}=\left[\begin{array}{ccc} +1 & +1 & +1\\ 0 & 0 & 0\\ -1 & -1 & -1 \end{array}\right]\;\mathrm{and}\;Gy_{\left(Prewitt\right)}=\left[\begin{array}{ccc} -1 & 0 & +1\\ -1 & 0 & +1\\ -1 & 0 & +1 \end{array}\right]$$

#### 3.2.4. Laplacian of Gaussian (LoG) Operator

A method was proposed where finding the zero-crossings in the 2nd derivative of the image intensity can detect the edge point in an image. Unfortunately, the 2nd derivative is very sensitive to noise. In this case, the noise should be filtered before edge detection. In order to achieve that, the LoG operator performs Gaussian smoothing before applying Laplacian [22].

In this method, the image is convolved with a Gaussian filter first. This step can smoothen the image and reduces noise. Since the width of the edge increases in the smoothing process, only the point having the local maximum value should be regarded as an edge. Therefore, the 2nd derivative operator, Laplacian, is used for this purpose. In order to reduce unnecessary edge pixels, only pixels whose first-order differential values (threshold) of zero-crossings exceed a certain degree are treated as edge points.

The output of the LoG operator: *h*(*x*, *y*) is obtained by the convolution operation:

(14) $$h(x,y)=\Delta^{2}\left[G(x,y)\times f(x,y)\right]=\left[\Delta^{2}G(x,y)\right]\times f(x,y)$$

where the following equation is normally called the Mexican hat operator.

(15) $$\Delta^{2}G_{\left(\log\right)}(x,y)={\left(\frac{x^{2}+y^{2}-2\sigma^{2}}{\sigma^{4}}\right)}^{-(x^{2}+y^{2})/2\sigma^{2}}$$

## 4. Experimental Study

### 4.1. Experimental Setup

**Figure 5:**
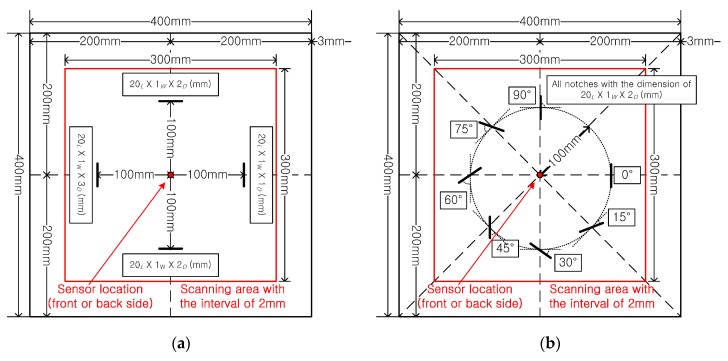
Configuration of notch damage on the aluminum plate (subscripts for the dimension of the notch, *L*, *W*, and *D* mean length, width, and depth, respectively), (**a**) different depth, (**b**) different angle.

**Figure 6:**
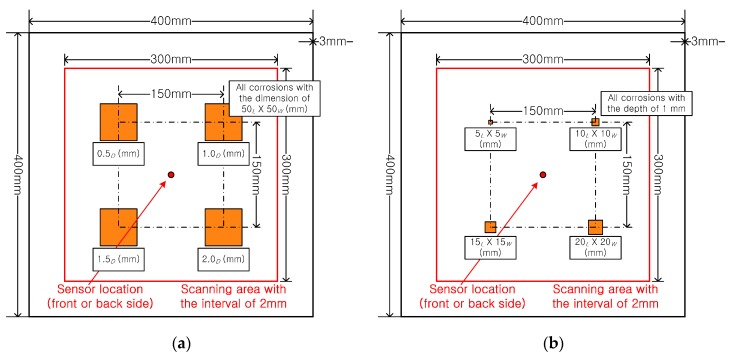
Configuration of corrosion damage on the aluminum plate (subscripts for the dimension of the notch, *L*, *W*, and *D* mean length, width, and depth, respectively), (**a**) different depth, (**b**) different size.

In this study, the 6061-T6 aluminum plates were selected as test specimens, which had dimensions of 400 × 400 mm with the thickness of 3 mm. After scanning the intact specimen, the notche and the corrosion damages were artificially formed on four specimens, as shown in Figure 5 and Figure 6. Figure 5a shows the designed condition of the first specimen; four notches were made as the same angles which are parallel to the tangent of wave front. This notch direction arrangement is used to verify the influence of the notch depth on the test results. Figure 5b shows the designed condition of the second specimen; seven artificial notches were formed on the plate, and the dimensions of each notch was 20 mm long, 1 mm long, and 2 mm deep. In addition, the direction of the notches is formed with a counter-clockwise increase of 15° for each notch, starting from the notch at the right area which is tangential to the wave front. This notch direction arrangement is used to verify the influence of the notch direction on the test results.

Next, corrosion damages were considered. The corrosion damages on the aluminum plates were artificially formed using concentrated hydrochloric acid, as shown in Figure 6. Figure 6a shows the designed condition of the third specimen, all corroded areas had the same size of 50 × 50 mm, but they had different depths with 0.5, 1.0, 1.5, and 2.0 mm. This arrangement is used to verify the influence of the depth of the corrosion on the test results. Figure 6b shows the designed condition of the forth specimen, and the corrosion areas had the same corrosion depth but different dimensions with 5 × 5, 10 × 10, 15 × 15, and 20 × 20 mm. This arrangement was used to verify the influence of the corroded area on the test results.

**Figure 7:**
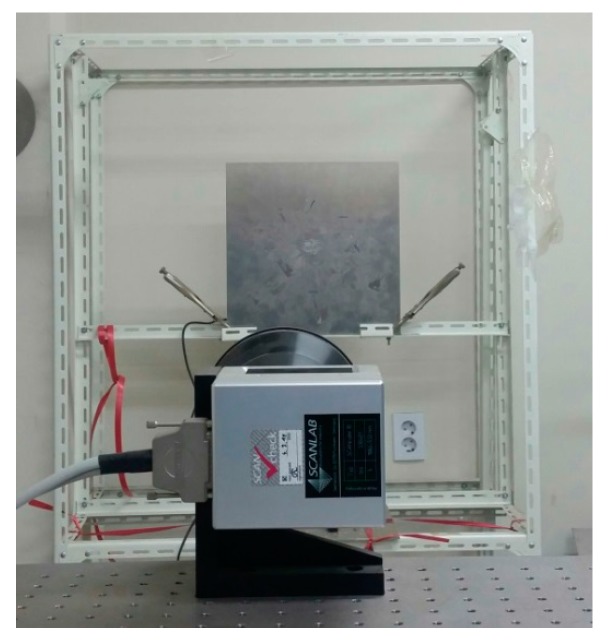
Location of the fixed target specimen during scanning.

Figure 7 shows that the specimen was fixed on a metal support. The bottom part of the specimen was tightly clamped with two clamps on the metal frame. In this study, the sensor was attached to the central position on the back side of the scanned surface. An amplifier-integrated acoustic emission (AE) sensor was used to measure the multiple wave signals. The AE sensor has a broadband characteristic with upper and lower cutoff frequencies of 2 MHz and 100 kHz, respectively. The resonant frequency of the sensor is 200 kHz ± 20%. The maximum sensitivity of the sensor is 120 ± 3 dB at the resonant frequency. The scanned area was 300 × 300 mm at the central part of the specimen; in this area, a 151 × 151 point grid can be generated, with the scanning interval of 2 mm. The distance between the laser mirror scanner and the target specimen was 2 m.

### 4.2. Comparison between RMS Images and Edge Detection Results

#### 4.2.1. Damage Case 1: Notch

**Figure 8:**
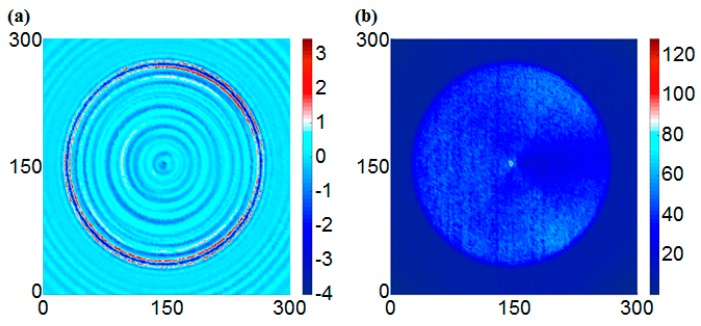
Snapshots of the intact specimen at 40 μs: (**a**) UWPI snapshot, (**b**) RMS (Root Mean Square) snapshot.

Figure 8 shows the scanning results of the intact specimen at 40 μs. For the UWPI snapshots, the wave packet propagated radially in a dispersed fashion from the central location in the circumferential boundary condition as shown in Figure 8a. Figure 8b shows the estimated RMS snapshots from Figure 8a. The results showed that the color of the scanned area is uniform; this means a structural condition of the smooth plate surface. Because more wave energy was accumulated in the vicinity of the sensor location at the early stage, the color was lighter than for the other areas; this means that bigger RMS values were estimated in the sensor location.

**Figure 9:**
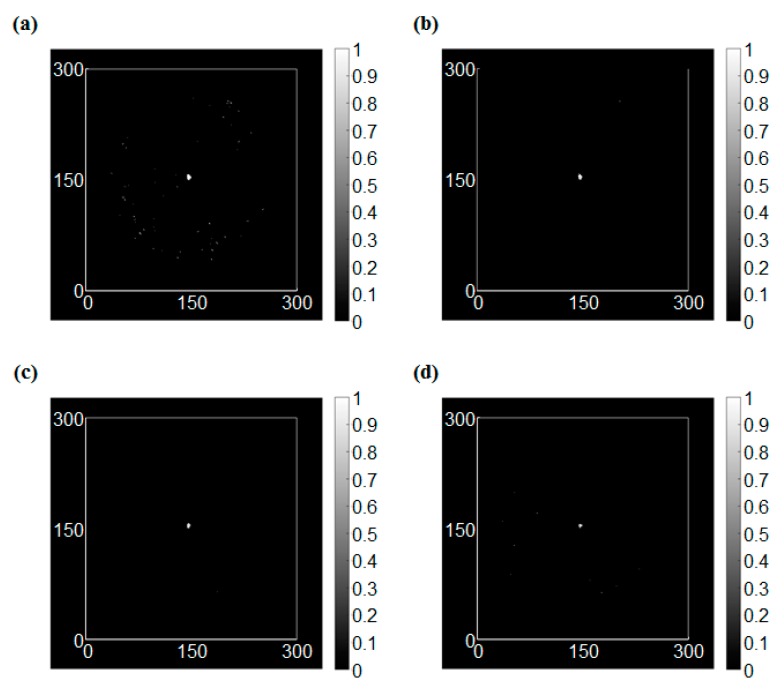
The edge detection results of the intact condition, (**a**) Sobel operator (threshold = 1.3), (**b**) Roberts cross operator (threshold = 0.3), (**c**) Prewitt operator (threshold = 1.4), (**d**) LoG (Laplacian of Gaussian) operator (threshold = 0.3).

The edge detection results for the intact condition are shown in Figure 9. In this study, four types of operators, which were Sobel, Roberts, Prewitt, and LoG operators, were applied to the RMS images. In this case, the RMS values were dramatically changed only at the vicinity of the sensor. On the other hand, the RMS values were varied smoothly at the boundary of the wave front. As a result, the edge was detected at the sensor location clearly for all operators.

**Figure 10:**
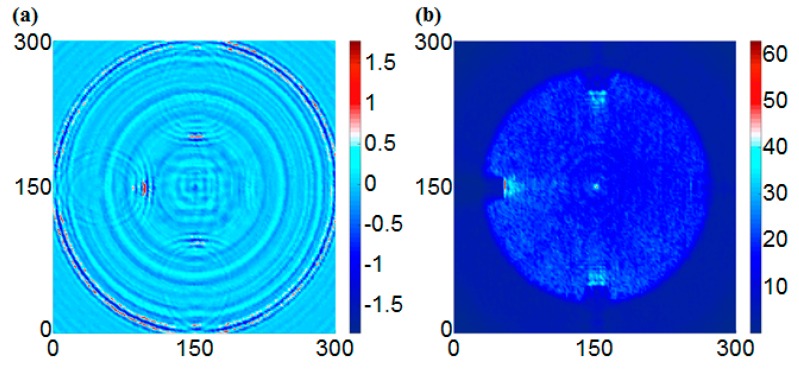
Snapshots of specimen 1 at 40 μs: (**a**) UWPI snapshot, (**b**) RMS snapshot.

Next, the variation in depth of the notch was considered. Figure 10a shows a UWPI snapshot at 40 μs, and anomalous wave due to the damage can be observed near the damage locations. Furthermore, the reflected wave became a source of new scattered waves when the propagating waves encountered the damage. The influence of the reflected waves at the right area (depth = 1 mm) was the lowest, and was also not significant. On the other hand, the influence of the reflected waves at two notches (depth = 2 mm) which are located in the upper and lower area can be more clearly observed than a notch at the right area (depth = 1 mm). Furthermore, the result at the left area (depth = 3 mm) was most significant. In Figure 10b, the results showed that the larger values occurred at reflected wave paths. Any reflected waves almost cannot be observed at the notch with the depth of 1 mm, but the other three notches were observed successfully because the RMS values are lowest at the shallowest notch. Because the two notches of the upper and lower parts have the same condition, their results were very similar, and the biggest RMS value was measured at the notch of 3 mm.

**Figure 11:**
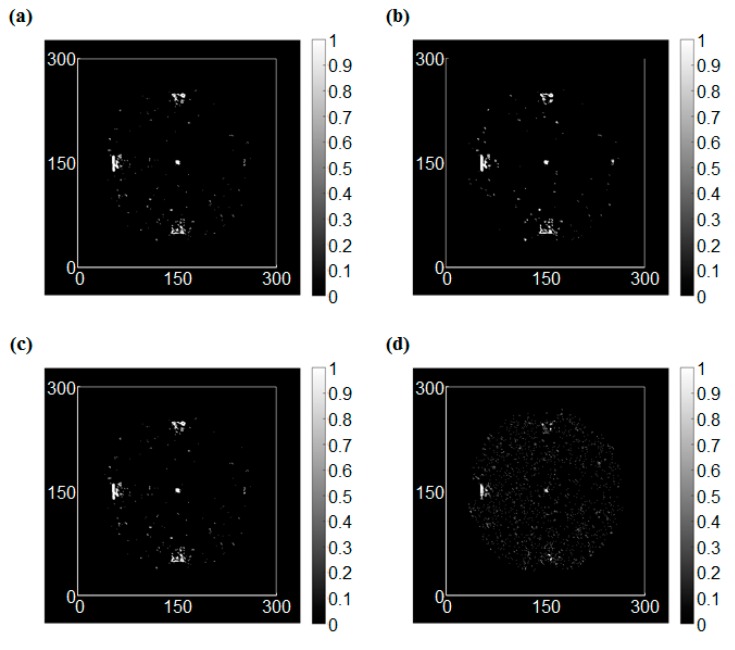
The edge detection results of the damaged condition with the different depth, (**a**) Sobel operator (threshold = 1.1), (**b**) Roberts cross operator (threshold = 0.2), (**c**) Prewitt operator (threshold = 0.8), (**d**) LoG operator (threshold = 0.15).

The edge detection process was applied again in this case as shown in Figure 11. The shallowest notch at the right side can be visualized, but it is not still clear, while it is hard to identify this notch through the RMS image. Unfortunately, however, the variation in the depth of the notches is not clearly expressed in the results of the edge detection operations although the edges of the notches are clearly detected. It means that the edge detection process may be appropriate for detecting the shape of the damage. On the contrary, the method has poor quality to detect the depth of the damage.

**Figure 12:**
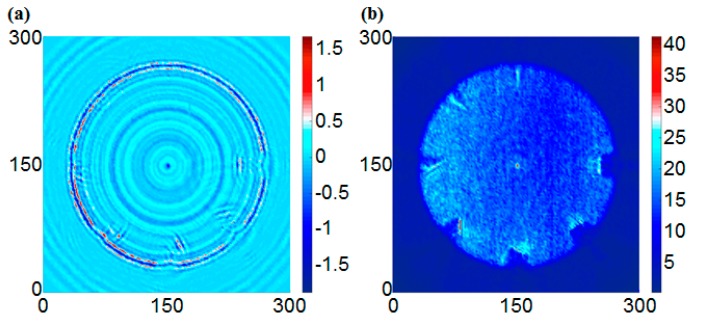
Snapshots of specimen 2 at 40 μs: (**a**) UWPI snapshot, (**b**) RMS snapshot.

The UWPI and RMS snapshots of the notches with different angles were captured at 40 μs and are shown in Figure 12. Since the waves propagate along the radial direction, the wave portions have stress components in the vertical and horizontal directions and hence the reflected waves can be observed regardless of the angle between the wave front and the notch, as shown in Figure 12b. In this case, the energy of the reflected waves is largest when the notch is tangential to the wave front. On the other hand, the smallest value of RMS is observed when the notch is perpendicular to the wave front. This is because the wider notch can reflect the incident waves.

**Figure 13:**
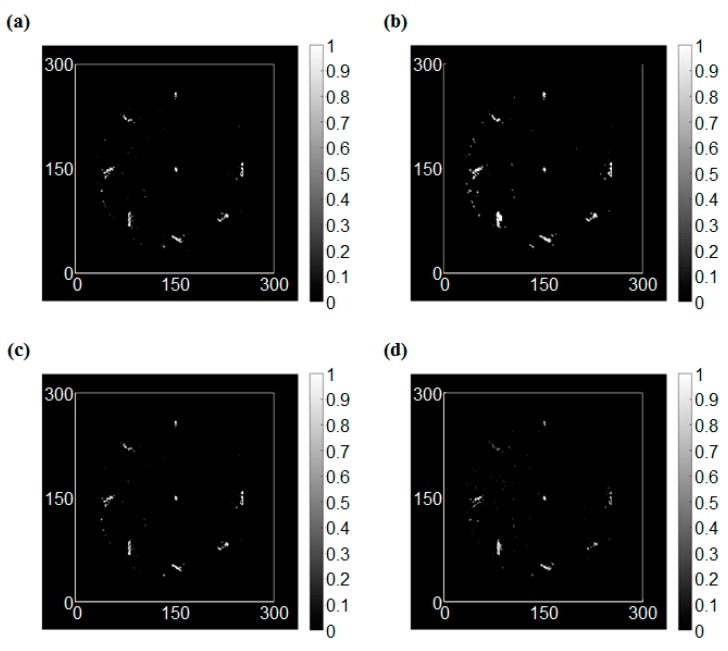
The edge detection results of the damaged condition with the different angle, (**a**) Sobel operator (threshold = 1.8), (**b**) Roberts cross operator (threshold = 0.3), (**c**) Prewitt operator (threshold = 1.4), (**d**) LoG operator (threshold = 0.3).

In this case, the edges of the notches were most clearly detected compared to the other case. Additionally, the noise near the notches is hardly observed because the depths of the notches were identical and the size of the notches was enough to reflect the incident waves. As mentioned previously, the edge detection is good at detecting the shape of the damage and hence the angles of the notches are clearly visualized using the edge detection process as shown in Figure 13.

#### 4.2.2. Damage Case 2: Corrosion

**Figure 14:**
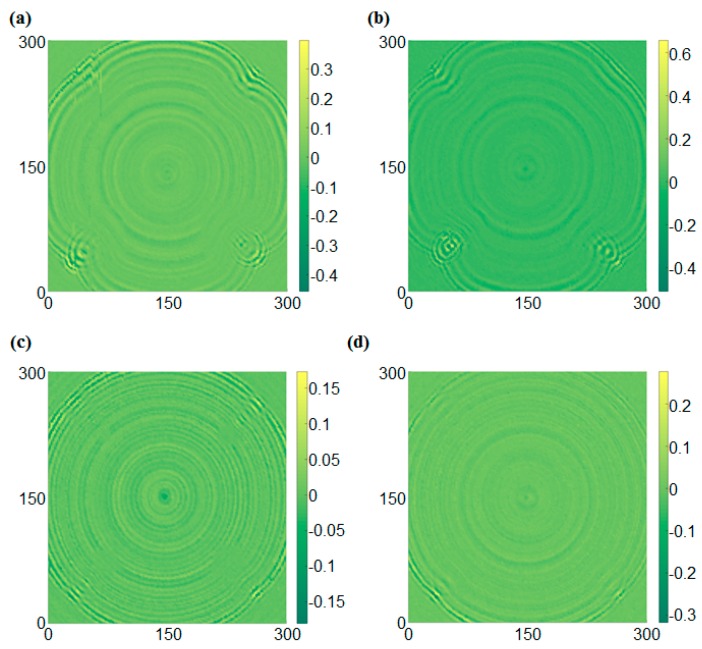
UWPI snapshots at 60 μs: (**a**) Front side of the first specimen, (**b**) Back side of the first specimen, (**c**) Front side of the second specimen, (**d**) Back side of the second specimen.

**Figure 15:**
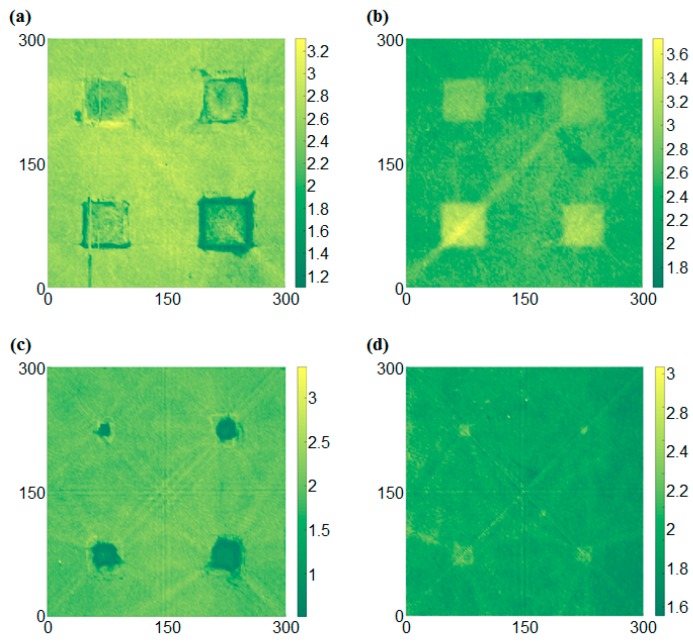
RMS snapshots at 400 μs: (**a**) Front side of the first specimen, (**b**) Back side of the first specimen, (**c**) Front side of the second specimen, (**d**) Back side of the second specimen.

Figure 14 shows the UWPI results of the two specimens, in which one includes corrosion with different depths and the corrosion with different areas is made on the other plate, at 60 μs. Figure 14a shows a front side result of the first specimen at 60 μs, and the damage-induced anomalous wave can be observed at the damage locations. Furthermore, the reflected wave became a source of new scattering waves when the propagating waves encountered the damage. The result in Figure 14a showed that the propagating waves were obviously scattered. At the top left area, the scattering influence was the smallest, and the corrosion at the top right was more significant. Additionally, the wave reflection phenomena occurred at the bottom left and bottom right areas, which at the area with greater depth was clearer. Therefore, these features can be used for damage detection. Figure 14b shows the back side result of the first specimen. The results were almost the same as the results obtained from the front side. Figure 14c,d shows the results from both sides of the second specimen. The scattering phenomenon also occurred at the corrosion areas, and the degrees of interference at both sides were similar. Figure 15 shows the RMS result of the two specimens. The results showed that the corrosion areas were observed clearly, the damage with deeper depth showed a deeper color on the front side, and a lighter color was shown at a deeper depth on the back side. Therefore the damage was successfully detected. In addition, the results show that the RMS method has a good ability for classification of the damage depth.

**Figure 16:**
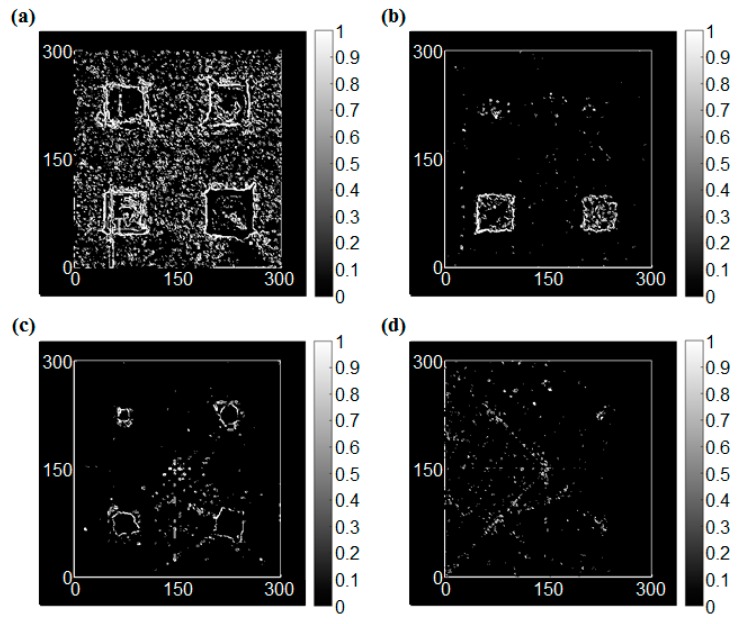
The edge detection results using the Sobel operator (threshold = 2); (**a**) Front side of the first specimen, (**b**) Back side of the first specimen, (**c**) Front side of the second specimen, (**d**) Back side of the second specimen.

**Figure 17:**
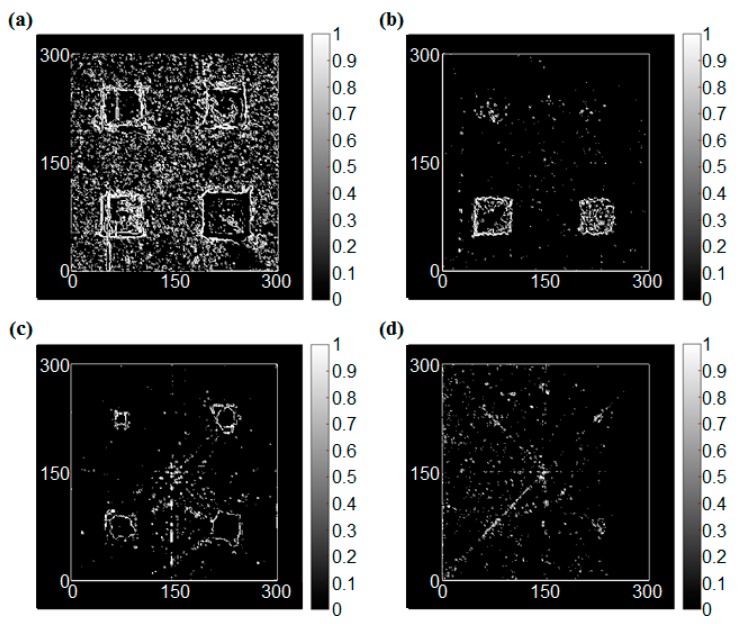
The edge detection results using the Roberts cross operator (threshold = 0.4); (**a**) Front side of the first specimen, (**b**) Back side of the first specimen, (**c**) Front side of the second specimen, (**d**) Back side of the second specimen.

**Figure 18:**
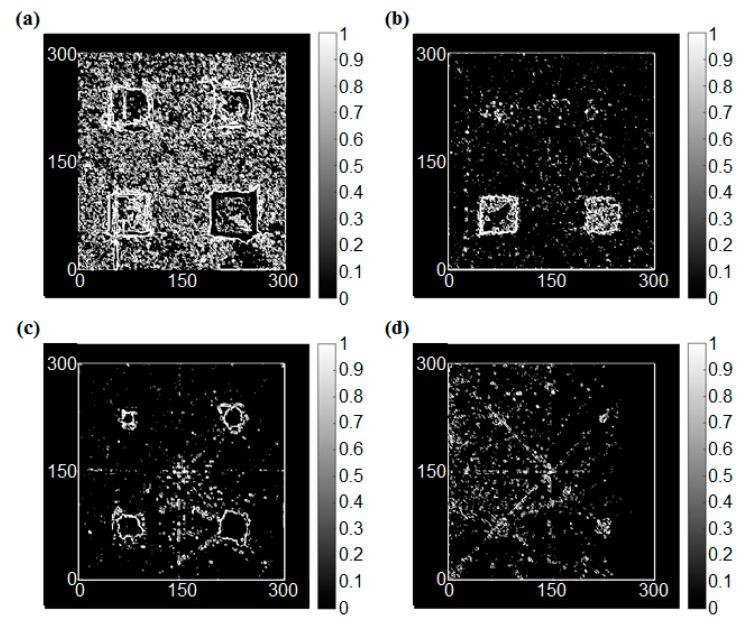
The edge detection results using the Prewitt operator (threshold = 1); (**a**) Front side of the first specimen, (**b**) Back side of the first specimen, (**c**) Front side of the second specimen, (**d**) Back side of the second specimen.

**Figure 19:**
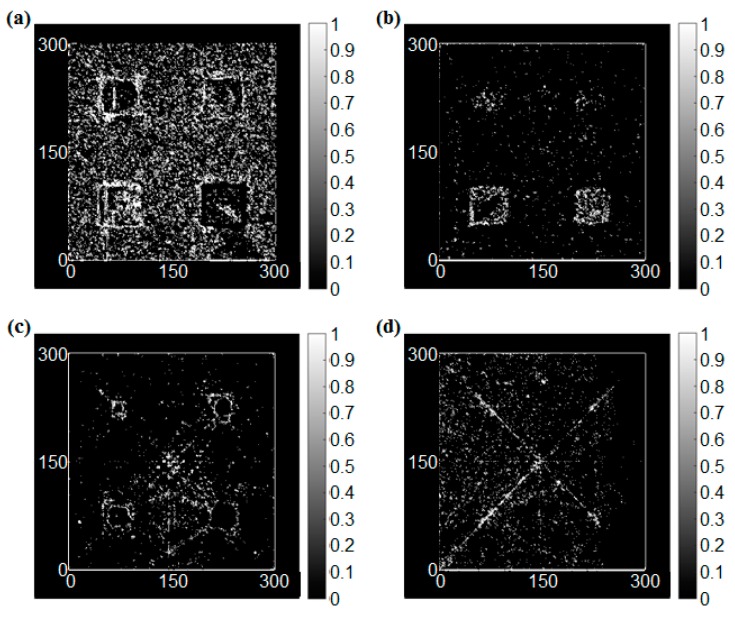
The edge detection results using the LoG operator (threshold = 0.3); (**a**) Front side of the first specimen, (**b**) Back side of the first specimen, (**c**) Front side of the second specimen, (**d**) Back side of the second specimen.

After edge detection processing, the grayscale images of Sobel, Roberts, Prewitt, and LoG operators are shown in Figure 16, Figure 17, Figure 18 and Figure 19, respectively. The color axis showed a standard of edge strength, in which a bigger RMS value has a more dramatic change of the gray-scale (strong edge), and it also can be considered as deeper damage in this study. In this case, the threshold of the Sobel, Roberts, Prewitt, and LOG operators were 2, 0.4, 1, and 0.3, respectively.

For the Sobel operator, Figure 16a shows a remarkable classification of the damage degrees. A deeper damage has a more dramatic change of the gray-scale (a bigger value). The result of Figure 16c shows a similar degree of edge with the same depths. For the back side, in Figure 16b the damages of 1.5 mm and 2 mm are observed clearly. However, because the depth of some damage is not deep enough (1 mm or less than 1 mm), it cannot be observed very clearly in Figure 16b,d. Compared with the Roberts and Prewitt operator, the results were very similar. It is worth noting that the effect of the Prewitt operator for a small area of corrosion damage detection was better, as shown in Figure 18c. This result means that the Prewitt operator is more sensitive to horizontal and vertical damage. However, the result of the LoG operator was not clearer than the other three operators, possibly due to the method further reducing the number of unnecessary pixels. On the other hand, the result in Figure 19d showed that the LoG operator is more sensitive to the diagonal edges. In summary, the results successfully detected the corrosion. In addition, the results from the measurement at the front side of the plate, where the corrosion damages were directly exposed to the laser beam, were better than the results from the back-side scanning. Additionally, the damage detection for the corrosion damages based on the edge detection method will not be as good as the RMS method.

## 5. Conclusions

In this study, notch and corrosion damage on aluminum plates were investigated using the UWPI imaging system, which utilizes a Q-switched Nd:YAG pulsed laser system and a laser scanner based on a galvanometer. First, an intact aluminum plate without any damage was scanned. In this case, the results showed continuities of the propagating waves in the snapshots at the raw UWPI, filtered UWPI, and RMS snapshots. Then, two conditions of notches were artificially formed, in which one was the notches with different depths and different angles were considered in the other condition. In the case of different depths of the notches, the reflected waves could be clearly visualized through the RMS calculation except for the shallowest notch. The energy of the reflection at the shallowest notch was relatively low compared to other notches because the depth which could reflect the incident waves was shallow. After the edge detection processing, the notches were clearly visualized even at the point of the shallowest notch. However, the differences in depth were hardly detected using the edge detection method. In the case of different angles of the notches, the damage was clearly detected in the RMS snapshots and in the images obtained from the edge detection process. Since the incident waves in this study propagated along the radial direction, the waves had stress components in the vertical and horizontal directions. Also, all of the notches had the same depth in this case. Therefore, the reflected waves were clearly observed regardless of the angles of the notches. However, the tangential notch to the wave front was most clearly detected while the energy of the notch perpendicular to the wave front was the lowest, because the reflected waves were affected by the width of the notch. Next, corrosion damage was considered in a similar manner with that of the notch damage. Also, in this case, the depth and the area of the corrosion damage were investigated. For both conditions, the scanning results obtained from the front side of the specimens were clearer than the back side of the specimens at the RMS and edge detection snapshots. In this case, the depth of the damage was hard to detect while the size of the damage could be clearly identified. As a result, the RMS snapshots are appropriate for observing the energy flow of the propagating wave while the image obtained from the edge detection method is good for distinguishing the shape of the damage. Unfortunately, however, the variation in the depth of the damage is hardly investigated using the edge detection method.
